# Impact of calcium administration on survival and neurologic outcomes in adults with in-hospital cardiac arrest

**DOI:** 10.1016/j.resplu.2026.101427

**Published:** 2026-07-24

**Authors:** Julia Hawthorne, Saman Razzaq, Chelsey Malhas, Yunhan Liao, Jie Yang, Sam Parnia, Sahar Ahmad, Jignesh K. Patel

**Affiliations:** a Department of Medicine, Stony Brook Renaissance School of Medicine, Stony Brook, NY, United States; b Biostatistical Consulting Core, Stony Brook Renaissance School of Medicine, Stony Brook, NY, United States; c Department of Medicine, New York University Grossman School of Medicine, New York, NY, United States

**Keywords:** Calcium, Cardiac arrest, In-hospital cardiac arrest, Survival, Mortality, Neurologic outcome, Outcome, Return of spontaneous circulation

## Abstract

**Background:**

Calcium administration during cardiac arrest has historically been used for its potential inotropic effects, despite limited evidence of benefit. Prior studies in out‑of‑hospital cardiac arrest suggest harm, but data specific to in‑hospital cardiac arrest (IHCA) remain limited.

**Methods:**

We conducted a retrospective cohort study of adult IHCA events occurring at a single academic tertiary‑care medical center in the United States between 2011 and 2024, identified from a prospective registry. IHCA events lasting at least 5 min were included. The primary exposure was intravenous calcium gluconate administration during resuscitation. The primary outcome was sustained return of spontaneous circulation (ROSC ≥20 min). Secondary outcomes were survival to hospital discharge and favorable neurological outcome at discharge (Glasgow Outcome Scale score 4–5). Multivariable logistic regression analyses were performed.

**Results:**

Among 599 IHCA events, 234 patients (39.1%) received calcium. Calcium administration was associated with significantly lower rates of sustained ROSC (31.6% vs 57.8%), survival to hospital discharge (6.8% vs 18.9%), and favorable neurological outcome (3.0% vs 14.3%; *p* < 0.0001 for all). After multivariable adjustment, calcium use remained associated with lower odds of sustained ROSC (OR 0.46; 95% CI 0.31–0.68), survival to hospital discharge (OR 0.36; 95% CI 0.19–0.68), and favorable neurological outcome (OR 0.20; 95% CI 0.08–0.49). No interaction was observed between calcium administration and CPR duration.

**Conclusion:**

Calcium administration during IHCA was independently associated with lower rates of sustained ROSC, survival to hospital discharge, and favorable neurological outcome. These findings support current guideline recommendations against routine calcium use during undifferentiated IHCA.

## Introduction

For the past century, calcium has been utilized during cardiac arrest due to its important role in myocardial excitation–contraction coupling, intracellular signaling, and inotropic support.^1^, ^2^, ^3^ The benefit of calcium administration in patients with cardiac arrest has become increasingly questioned over the past four decades.^4^, ^5^, ^6^, ^7^ Studies assessing prognostic outcomes with calcium administration during cardiac arrest have yielded conflicting results.^8^, ^9^, ^10^, ^11^, ^12^, ^13^, ^14^, ^15^, ^16^ The 2021 Calcium for Out-of-hospital Cardiac Arrest (COCA) randomized trial was halted early due to concerns that calcium administration was associated with a lower likelihood of achieving return of spontaneous circulation (ROSC) and worse long-term neurologic outcomes – but noted to have wide confidence intervals.^10^, ^11^, ^12^ Multiple systematic reviews have failed to show evidence that routine calcium administration improves the outcomes of cardiac arrest in adults or children.^17^, ^18^, ^19^

The 2025 American Heart Association and European Resuscitation Council guidelines currently recommend against routine administration of calcium for treatment of cardiac arrest, reserving its use for select indications such as hyperkalemia, hypocalcemia, or calcium channel blocker toxicity.^20^, ^21^, ^22^ The 2023 International Liaison Committee on Resuscitation (ILCOR) review on this topic also advised against the routine administration of calcium for treatment of in-hospital and out-of-hospital cardiac arrest.^23^ Despite these recommendations, calcium continues to be frequently administered during IHCA, with prior registry data demonstrating increasing use over time.^24^

Given the persistence of calcium administration during IHCA and the limited contemporary outcome data specific to this setting, we sought to evaluate the association between calcium administration and clinically meaningful outcomes, including sustained ROSC, survival to hospital discharge, and favorable neurological outcome at discharge.

## Methods

### Study population and data collection

We performed a retrospective cohort study of adult patients (≥18 years) undergoing resuscitation for IHCA at a single academic tertiary-care medical center in the United States between 2011 and 2024, identified from a prospective institutional registry. IHCA events lasting at least 5 min were included. Cardiac arrests with CPR duration of less than 5 min were excluded because these represent very brief resuscitation events in which there is likely insufficient time for calcium to have been administered and to exert a meaningful physiological effect. Arrests occurring between January 2020 and December 2023 were excluded due to COVID-19–related alterations in resuscitation protocols. Arrests occurring in the emergency department or surgical intensive care units were also excluded.

The primary exposure was administration of intravenous calcium gluconate during IHCA, identified from code documentation in the electronic medical record. Calcium gluconate was the only calcium salt included in this study as it was the sole calcium salt available at the study site. Patients were categorized as receiving calcium or no calcium during resuscitation. The primary outcome was sustained ROSC, defined as ROSC lasting at least 20 min. Secondary outcomes were survival to hospital discharge and favorable neurological outcome at discharge, defined as a Glasgow Outcome Scale (GOS) score of 4 or 5. Although Cerebral Performance Category (CPC) is the most commonly reported neurologic outcome measure in cardiac arrest research, GOS assesses a similar construct of global neurologic function and disability, and favorable outcomes defined by GOS score or 4–5 are generally considered comparable to favorable outcomes defined by CPC score of 1–2.^25^

Patient demographics, comorbidities, cardiac arrest characteristics (including initial rhythm, arrest location, bicarbonate administration, time to first epinephrine dose, and CPR duration), and laboratory data were extracted from the medical record. Race was obtained from the electronic health record, where they are recorded based on patient self-report at registration. Race categories in the EHR included White, Black or African American, Asian, American Indian or Alaska Native, Native Hawaiian or Other Pacific Islander, Other, and Unknown. Hyperkalemia was defined as a serum potassium value ≥5.5 mEq/L measured within 24 h prior to arrest. Renal disease included documented moderate-to-severe chronic kidney disease or acute renal failure.

### Statistical analysis

Chi-square tests with exact *p*-values based on Monte Carlo simulation were utilized to examine the marginal associations between categorical variables (patients’ characteristics, medical conditions, clinical outcomes) and calcium administration (Calcium vs. No Calcium) or between categorical variables and clinical outcomes (ROSC, survival to hospital discharge, neurological outcome at discharge). Wilcoxon rank sum tests were utilized to compare the unadjusted marginal differences in continuous variables (age, time to first epi in minutes) by calcium administration and clinical outcomes.

CPR duration was reported as a continuous variable for descriptive statistics only. Data on timing of calcium administration was not available within our dataset. Thus, to evaluate whether the association between calcium administration and outcomes changes across CPR duration categories, CPR duration was adjusted for as a binary variable and used to test the interaction between calcium administration and binary CPR duration. Patients were stratified by CPR duration (5–8 min and >8 min). These categories were chosen a priori to distinguish shorter versus prolonged resuscitation. Additionally, because the study population was predominantly White (approximately 80%), race was dichotomized as White vs. non-White for univariable analysis and inclusion in the multivariable model due to insufficient sample sizes in individual minority racial categories to support stable effect estimates. Race was included in the analysis to assess potential disparities in clinical outcomes and to evaluate the generalizability of findings.

Calcium administration, duration of CPR, and their interaction term were included in the multivariable logistic regression model to investigate whether the different risk of having ROSC, surviving to hospital discharge, or having a good neurological outcome at discharge between patients with and without calcium administration was different across different duration of CPR categories. Other factors that were significantly related to ROSC based on univariate analyses with *p*-value < 0.05 were adjusted.

Based on the 10-events-per-variable rule, the number of covariates that could be included in the models for secondary outcomes was limited.^26^ We therefore used outcome-specific models for these two outcomes; covariates were selected based on both their significant associations with the respective outcomes in univariate analyses and their clinical relevance. Variables were prioritized based on likelihood to influence calcium administration in the clinical setting: hyperkalemia and renal disease (given risk of hyperkalemia and hypocalcemia in patients with renal disease). For survival to hospital discharge, the model was adjusted for age, sex, hyperkalemia, renal disease, and non-shockable initial rhythm. For neurological outcome at discharge, the model was adjusted for hyperkalemia, renal disease. Firth's correction was applied to mitigate the bias caused by rare events in surviving to hospital discharge or having a good neurological outcome at discharge for calcium administered patients who had duration of CPR of 5–8 min.^27^

Furthermore, a model excluding duration of CPR was examined in the multivariable logistic regression model to assess the effect of calcium administration alone. For ROSC, the model was adjusted for the same covariates. For survival to hospital discharge, the model was adjusted for age, sex, hyperkalemia, renal disease, non-shockable rhythm, and race. For neurological outcome at discharge, the model was adjusted for hyperkalemia, renal disease, and non-shockable rhythm. In logistic regression models, an odds ratio (OR) >1 indicated a higher risk of having the outcomes (ROSC, survival to hospital discharge) or a good neurological outcome at discharge, while an OR <1 indicated a lower risk. Statistical analyses were performed using SAS 9.4 (SAS Institute Inc., Cary, NC) and significance level was set at 0.05.

## Results

**Fig. 1:**
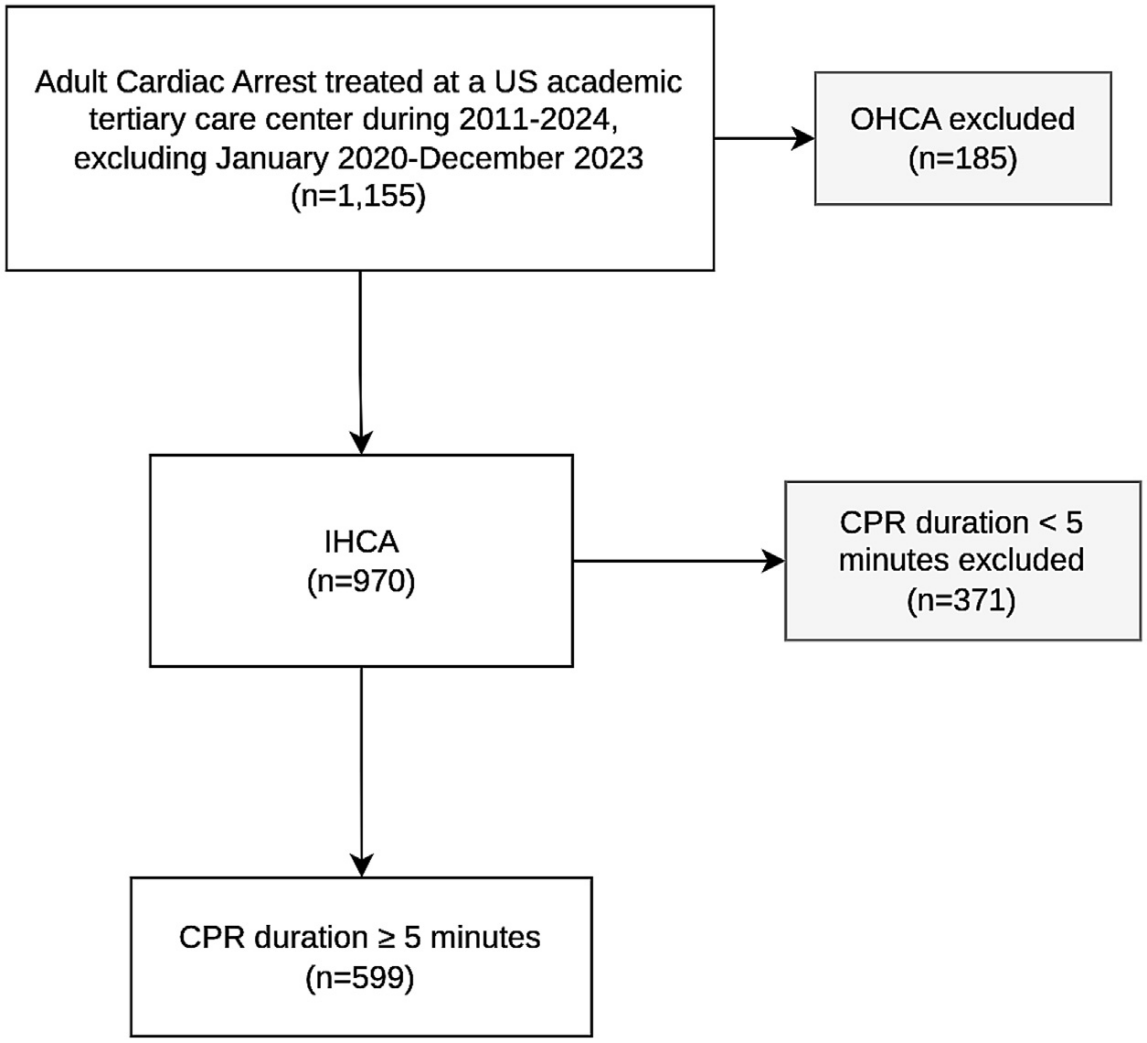
**Flowchart detailing total cardiac arrests and subsequent exclusions**.

A total of 599 adult in-hospital cardiac arrest (IHCA) events met inclusion criteria. Fig. 1 illustrates the selection of patients included in the analysis. Among these, 234 patients (39.1%) received intravenous calcium during resuscitation, while 365 patients (60.9%) did not. Baseline comorbid conditions were generally similar between exposure groups and are summarized in Table 1. Patients who received calcium more frequently had underlying renal disease, hyperkalemia, leukemia, and mild liver disease.

**Table 1:** Baseline comorbidities by exposure group.

**Medical history, No. (%)**	**Calcium (*n* = 234)**	**No calcium (*n* = 365)**	*P*-value*
Arterial hypertension	153 (65.4%)	241 (66.0%)	0.9293
Coronary artery disease	98 (41.9%)	157 (43.0%)	0.8008
Congestive heart failure	91 (38.9%)	146 (40.0%)	0.7998
Heart attack (myocardial infarction)	43 (18.4%)	51 (14.0%)	0.1585
Hypercholesterolemia	110 (47.0%)	175 (48.0%)	0.8694
Arrhythmia (e.g. atrial fibrillation)	68 (29.1%)	113 (31.0%)	0.6435
Chronic pulmonary disease	53 (22.7%)	108 (29.6%)	0.0737
Cerebrovascular disease (TIA, stroke)	27 (11.5%)	44 (12.1%)	0.8964
Thrombosis (PVT/PE)	22 (9.4%)	38 (10.4%)	0.7770
Diabetes mellitus	92 (39.3%)	133 (36.4%)	
Diabetes (without end-organ damage)	50 (21.4%)	77 (21.1%)	1.0000
Diabetes (with end-organ damage)	42 (18.0%)	56 (15.3%)	0.4181
Renal disease (all)	145 (62.0%)	192 (52.6%)	0.0264
Renal disease (moderate to severe)	78 (33.3%)	112 (30.7%)	
Acute renal failure	106 (45.3%)	133 (36.4%)	
Mild liver disease	14 (6.0%)	8 (2.2%)	0.0242
Moderate to severe liver disease	13 (5.6%)	17 (4.7%)	0.6941
Cancer (all)	68 (29.1%)	98 (26.9%)	
Solid tumor (non-metastatic)	28 (12.0%)	48 (13.2%)	0.7099
Malignant lymphoma	6 (2.6%)	12 (3.3%)	0.6271
Leukemia	19 (8.1%)	10 (2.7%)	0.0036
Metastatic solid tumor	15 (6.4%)	28 (7.7%)	0.6223
Dementia	11 (4.7%)	17 (4.7%)	1.0000

* *P*-values were based on Chi-squared test with exact *p*-value from Monte Carlo simulation.

Cardiac arrest characteristics were largely comparable between groups with respect to age, sex, initial arrest rhythm, and arrest location (Table 2). However, patients receiving calcium were more likely to receive bicarbonate during resuscitation. Median CPR duration was significantly longer among calcium recipients, and CPR duration exceeding 8 min occurred substantially more often in the calcium group.

**Table 2:** Cardiac arrest characteristics by exposure group.

Variable*	**Calcium (*n* = 234)**	**No calcium (*n* = 365)**	*P*-value**
Age (years)	69.0 ± 22.0	70.0 ± 19.0	0.3233
Sex			0.7358
Male	147 (62.8%)	223 (61.1%)	
Female	87 (37.2%)	142 (38.9%)	
Race			0.0228
Caucasian	197 (84.2%)	279 (76.4%)	
Other	37 (15.8%)	86 (23.6%)	
Initial rhythm			0.253
Ventricular fibrillation	15 (6.4%)	31 (8.5%)	
Ventricular tachycardia	18 (7.7%)	34 (9.3%)	
PEA	133 (56.8%)	206 (56.4%)	
Asystole	52 (22.2%)	68 (18.6%)	
Perfusing rhythm	1 (0.4%)	0 (0.0%)	
Bradycardia	9 (3.9%)	16 (4.4%)	
Unknown/undocumented	6 (2.6%)	10 (2.7%)	
Duration of CPR***			<.0001
Median (IQR)	26.5 ± 21	15 ± 17	
5–8 min, No. (%)	9 (3.9%)	86 (23.6%)	
>8 min, No. (%)	225 (96.2%)	279 (76.4%)	
Location of cardiac arrest			0.0528
General medical floors	104 (44.4%)	192 (52.6%)	
ICU	130 (55.6%)	173 (47.4%)	
Bicarbonate	204 (87.2%)	168 (46.0%)	<.0001
Hyperkalemia	51 (22.3%)	46 (13.1%)	0.0045

* For categorical variables, column percentages were reported; for continuous variables, median ± IQR were reported.

** For categorical variables, *p*-values were based on Chi-squared test with exact *p*-value from Monte Carlo simulation; for continuous variables, *p*-values were from Wilcoxon rank sum test.

*** For duration of CPR, *p*-value based on binary variable.

### Sustained return of spontaneous circulation

**Fig. 2:**
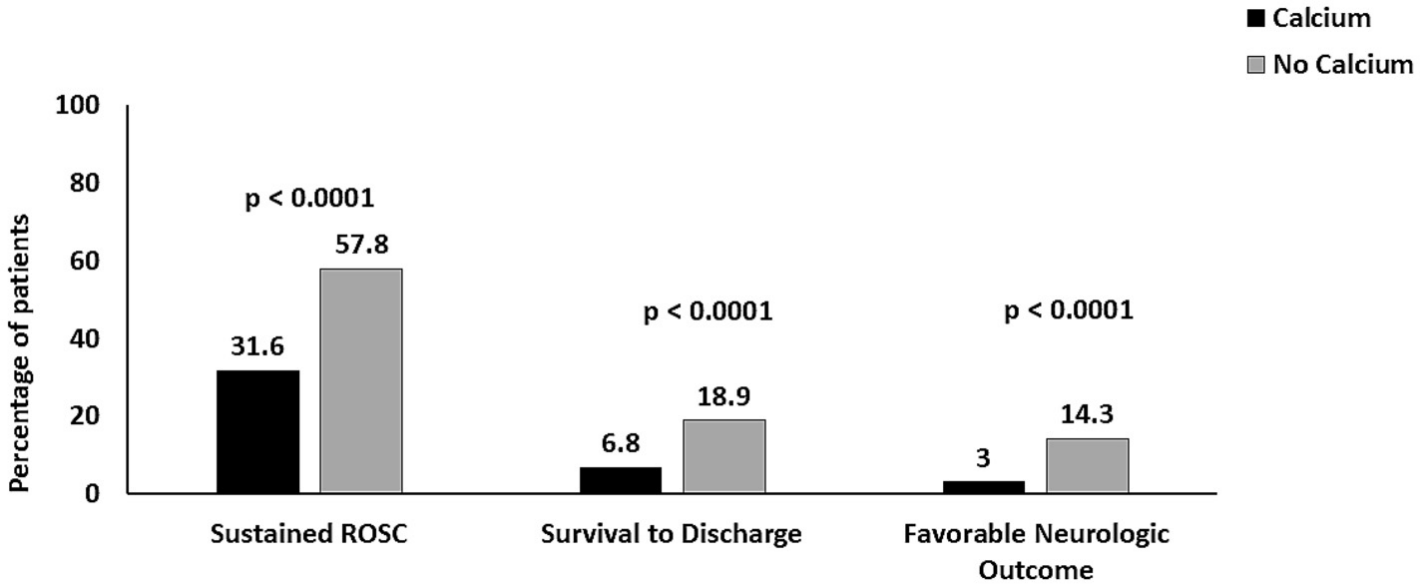
**Rates of sustained return of spontaneous circulation, survival to discharge, and favorable neurologic outcome with calcium versus no calcium administration**.

Overall, sustained return of spontaneous circulation (ROSC ≥20 min) was achieved in 285 patients (47.6%). Patients receiving calcium had significantly lower rates of sustained ROSC compared with those not receiving calcium (31.6% vs 57.8%, *p* < 0.0001) (Fig. 2). In multivariable logistic regression analysis adjusting for relevant confounders, calcium administration was independently associated with lower odds of sustained ROSC (OR 0.46; 95% CI 0.31–0.68; *p* < 0.0001) (Table 3). In models evaluating CPR duration as an effect modifier, calcium administration was associated with lower odds of ROSC among patients with CPR duration >8 min, while no significant association was observed among patients with CPR duration 5–8 min; no statistically significant interaction between calcium exposure and CPR duration was detected (interaction *p* = 0.85).

**Table 3:** Estimate OR and 95% CI of explanatory variables based on multivariable logistic regression model.

**Level**	**OR (95% CI)**	*P*-value*
**Sustained ROSC**		
Calcium administration	0.46 (0.31, 0.68)	<0.0001
Race (Caucasian vs other)	0.57 (0.36, 0.88)	0.0111
CA location (GMF vs ICUs)	1.29 (0.91, 1.84)	0.1592
Bicarbonate (no vs bicarbonate)	2.09 (1.38, 3.18)	0.0022
Hyperkalemia (no vs yes)	1.25 (0.78, 2.03)	0.3562

**Survival to hospital discharge**		
Calcium administration	0.36 (0.19, 0.68)	0.0015
Age	0.98 (0.97, 1.00)	0.0224
Sex (female vs male)	0.44 (0.24, 0.79)	0.0064
Hyperkalemia (no vs yes)	3.75 (1.12, 12.53)	0.0317
Renal disease (no vs yes)	2.10 (1.23, 3.58)	0.0064
Shockable vs non-shockable initial rhythm	1.76 (0.94, 3.30)	0.0795
Race (Caucasian vs other)	0.43 (0.24, 0.78)	0.0053

**Favorable neurological outcome at discharge**		
Calcium administration	0.20 (0.08, 0.49)	0.0003
Hyperkalemia (no vs yes)	3.70 (0.86, 15.90)	0.0786
Renal disease (no vs yes)	1.53 (0.84, 2.79)	0.1617
Shockable vs non-shockable initial rhythm	1.87 (0.95, 3.70)	0.0708

* *P*-value was from Type 3 analysis based on a multivariable logistic regression model.

### Survival to hospital discharge

Eighty-five patients (14.2%) survived to hospital discharge. Survival was significantly lower among patients receiving calcium compared with those who did not (6.8% vs 18.9%, *p* < 0.0001) (Fig. 2). After multivariable adjustment, calcium administration remained independently associated with lower odds of survival to hospital discharge (OR 0.36; 95% CI 0.19–0.68; *p* = 0.0015) (Table 3). Analysis stratified by CPR duration demonstrated persistently lower odds of survival among calcium recipients with CPR duration >8 min, while no survivors were observed among calcium-treated patients with CPR of 5–8 min. No significant interaction between calcium administration and CPR duration was identified (interaction *p* = 0.45).

### Favorable neurological outcome at discharge

A favorable neurological outcome at hospital discharge (Glasgow Outcome Scale score 4–5) was achieved in 59 patients (9.9%). Patients receiving calcium had significantly lower rates of favorable neurological outcome compared with those not receiving calcium (3.0% vs 14.3%, *p* < 0.0001) (Fig. 2). In adjusted analyses, calcium administration was independently associated with substantially lower odds of favorable neurological outcome at discharge (OR 0.20; 95% CI 0.08–0.49; *p* = 0.0003) (Table 3). When accounting for CPR duration, calcium use was associated with lower odds of favorable neurological outcome among patients with CPR duration >8 min. No patients receiving calcium with CPR of 5–8 min achieved a favorable neurological outcome, and no interaction between calcium administration and CPR duration was observed (interaction *p* = 0.66).

## Discussion

In this retrospective cohort of 599 adult in-hospital cardiac arrest (IHCA) events, administration of intravenous calcium during resuscitation was independently associated with worse clinical outcomes, including lower risk of sustained ROSC, survival to hospital discharge, and favorable neurological outcome at discharge. Multivariable logistic regression models testing for differences between calcium administration levels across CPR duration categories also showed that the calcium exposure group had significantly lower rates of sustained ROSC, survival to hospital discharge, and a favorable neurological outcome at discharge compared to those who did not receive calcium at CPR duration >8 min. Similar associations were found at CPR duration of 5–8 mins, but the results were no longer significant. When testing for differences between calcium administration levels excluding CPR duration, patients with calcium administration also had significantly lower risk of sustained ROSC, survival to hospital discharge, and a favorable neurological outcome at discharge compared to those without. These associations remained consistent in the sensitivity analyses. The uniform direction and magnitude of effect across multiple clinically meaningful endpoints strengthens the plausibility of a true adverse association between calcium administration and IHCA outcomes.

Our findings are concordant with emerging evidence from randomized trials and observational studies evaluating calcium administration during cardiac arrest.^8^, ^9^, ^10^, ^11^, ^12^, ^13^, ^14^, ^15^, ^16^ The COCA randomized clinical trial demonstrated no benefit of calcium administration and was terminated early due to concerns for potential harm, with lower rates of sustained ROSC in the calcium group compared with placebo.^10^, ^11^, ^12^ Subsequent observational studies in out-of-hospital cardiac arrest have similarly demonstrated decreased rates of ROSC or survival associated with calcium use, even after adjustment for confounding variables.^8^, ^9^, ^13^, ^16^ A recent systematic review conducted by the International Liaison Committee on Resuscitation (ILCOR) concluded that there is no evidence that routine calcium administration improves survival or neurological outcomes in adult cardiac arrest and emphasized the high risk of confounding by indication in observational analyses.^23^ Evidence specific to IHCA has been limited. Our study adds important contemporary data by examining a large IHCA cohort over more than a decade, demonstrating consistent adverse associations across resuscitation, survival, and neurological outcomes.

Importantly, although patients receiving calcium in our cohort more frequently had hyperkalemia and renal disease, adjustment for these conditions did not attenuate the association between calcium administration and worse outcomes. Furthermore, we observed no interaction between calcium administration and CPR duration, suggesting that differences in resuscitation length or arrest intensity alone do not explain the observed associations. These findings reinforce the principle that calcium should not be administered empirically during undifferentiated IHCA and should be reserved for clearly defined indications.

Several well-described physiological mechanisms provide biological plausibility for the observed association between calcium administration and worse outcomes. During global ischemia, ATP depletion and intracellular acidosis drive cytosolic calcium accumulation via Na^+^/H^+^ exchange and reversal of the Na^+^/Ca^2+^ exchanger, promoting hypercontracture and contraction band necrosis. Upon reperfusion, oscillatory calcium release activates calpains, phospholipases, and mitochondrial permeability transition pores, leading to mitochondrial dysfunction, reactive oxygen species generation, and irreversible cardiomyocyte injury. These calcium-mediated processes have been implicated as primary drivers of myocardial stunning and post-resuscitation myocardial dysfunction.^28^ Administration of exogenous calcium during CPR may exacerbate this calcium overload when myocardial cells are most vulnerable, worsening contractile dysfunction and impairing post-ROSC hemodynamic stability, while calcium's inotropic and vasopressor effects may increase myocardial oxygen demand during a low-flow state, potentially worsening ischemic injury. Similar calcium-mediated mechanisms have been implicated in cerebral ischemia–reperfusion injury,^29^ which may partially explain the markedly lower rates of favorable neurological outcome observed in this study.

This study has important limitations. As with all observational studies, residual confounding and confounding by indication cannot be fully excluded. Patients receiving calcium had a greater burden of comorbid illness, longer CPR durations, and higher rates of metabolic derangements, suggesting a sicker patient population. However, the persistence of the association between calcium administration and worse outcomes after multivariable adjustment and sensitivity analyses reduces the likelihood that confounding alone accounts for the magnitude and consistency of the findings. Additional limitations include the single-center design, lack of data on calcium dose, timing, or ionized calcium levels during resuscitation, and the inability to identify the precise clinical indication for calcium administration in each case. Neurological outcomes were assessed at hospital discharge and may not fully capture longer-term functional recovery. Nonetheless, discharge neurological status remains a clinically meaningful and patient-centered outcome in resuscitation research.

## Conclusion

In a contemporary cohort of adult IHCA events, calcium administration during resuscitation was independently associated with lower rates of sustained ROSC, survival to hospital discharge, and favorable neurological outcome. These findings reinforce current guidelines which recommend against routine calcium administration during undifferentiated IHCA and recommend the consideration of calcium administration for established indications.

## Funding

Stony Brook University Department of Medicine.

## CRediT authorship contribution statement

**Julia Hawthorne:** Writing – original draft, Data curation. **Saman Razzaq:** Data curation. **Chelsey Malhas:** Data curation. **Yunhan Liao:** Writing – review & editing, Writing – original draft, Visualization, Formal analysis. **Jie Yang:** Writing – review & editing, Writing – original draft, Visualization, Formal analysis. **Sam Parnia:** Writing – review & editing. **Sahar Ahmad:** Writing – review & editing, Writing – original draft, Methodology, Investigation, Conceptualization. **Jignesh K. Patel:** Writing – review & editing, Writing – original draft, Visualization, Validation, Supervision, Software, Resources, Project administration, Methodology, Investigation, Funding acquisition, Formal analysis, Data curation, Conceptualization.

## Declaration of competing interest

The authors declare that they have no known competing financial interests or personal relationships that could have appeared to influence the work reported in this paper.
